# Genome-wide identification of Aux/IAA gene family in white clover (*Trifolium repens* L.) and functional verification of *TrIAA18* under different abiotic stress

**DOI:** 10.1186/s12870-024-05034-3

**Published:** 2024-04-29

**Authors:** Tiangang Qi, Weiqiang Yang, Muhammad Jawad Hassan, Jiefang Liu, Yujiao Yang, Qinyu Zhou, Hang Li, Yan Peng

**Affiliations:** College of Grassland Science and Technology, Sichuan Agricultural University, Chengdu, 611130 China

**Keywords:** White clover, IAA gene family, Abiotic stress response, Functional verification

## Abstract

**Background:**

White clover (*Trifolium repens* L.) is an excellent leguminous cool-season forage with a high protein content and strong nitrogen-fixing ability. Despite these advantages, its growth and development are markedly sensitive to environmental factors. Indole-3-acetic acid (IAA) is the major growth hormone in plants, regulating plant growth, development, and response to adversity. Nevertheless, the specific regulatory functions of *Aux/IAA* genes in response to abiotic stresses in white clover remain largely unexplored.

**Results:**

In this study, we identified 47 *Aux/IAA* genes in the white clover genome, which were categorized into five groups based on phylogenetic analysis. The *TrIAAs* promoter region co-existed with different cis-regulatory elements involved in developmental and hormonal regulation, and stress responses, which may be closely related to their diverse regulatory roles. Collinearity analysis showed that the amplification of the *TrIAA* gene family was mainly carried out by segmental duplication. White clover *Aux/IAA* genes showed different expression patterns in different tissues and under different stress treatments. In addition, we performed a yeast two-hybrid analysis to investigate the interaction between white clover Aux/IAA and ARF proteins. Heterologous expression indicated that *TrIAA18* could enhance stress tolerance in both yeast and transgenic *Arabidopsis thaliana*.

**Conclusion:**

These findings provide new scientific insights into the molecular mechanisms of growth hormone signaling in white clover and its functional characteristics in response to environmental stress.

**Supplementary Information:**

The online version contains supplementary material available at 10.1186/s12870-024-05034-3.

## Background

Auxin, as an important endogenous plant hormone, is involved in various biological processes, such as plant growth and development, fruit ripening, formation of phototropism, and abiotic stress response [1, 2]. In plants, the gene family for early auxin response consists mainly of *Aux/IAA* (*Auxin/Indole-3-Acetic Acid*), *GH3* (*Gretchen Hagen3*), and *SAUR* (*SMALL AUXIN UP RNA*) [3]. Aux/IAA is a group of plant-specific, short-lived proteins located in the cell nucleus that function as transcriptional repressors in the auxin signaling pathway [4, 5]. The first Aux/IAA family member was isolated from soybeans (*Glycine max*) and subsequently identified in wheat (*Triticum aestivum* L.), rice (*Oryza sativa* L.), tomato (*Solanum lycopersicum*), and other crops [6–9]. Generally, the Aux/IAA protein consists of four protein-conserved domains, I, II, III, and IV, of which domain I contains the conserved motif ‘LxLxL’, which can interact with the TPL family of co-suppressors (TOPLESS) to form a co-repressor [10–12]. Domain II contains a conserved "GWPPV" motif that plays a role in the ubiquitination and degradation of Aux/IAA proteins by interacting with SCF^TIR1^ [13]. Domains III and IV are homologous to PB1, the C-terminal domain of the ARF protein, and can interact with ARFs to repress the expression of auxin-responsive genes [14]. In addition, there are truncated Aux/IAA proteins that do not have domain III or domain IV, such as the *CpIAA11*, *CpIAA19*, *CpIAA27*, and *CpIAA31* proteins in papaya (*Carica papaya* L.) [15].

In the auxin signaling pathway, auxin receptor TIR1/AFB (Transport Inhibitor Resistant/Auxin Signaling F-Box), transcriptional repressor Aux/IAA, and plant auxin response factor (Auxin Response Factor, ARF) are the three types of regulators [16, 17]. In the absence or at low auxin concentrations, the Aux/IAA protein interacts with the ARF protein through a homologous structural domain at the C-terminus, thus hinders the direct transcriptional regulatory effects of ARF on auxin-responsive genes [18–20]. Under high auxin levels, auxin binds to the receptor TIR1/AFBs, which makes it easy for TIR1 to bind with Aux/IAA proteins, and induces SCF^TIR1^-mediated ubiquitination degradation of Aux/IAA proteins [21, 22]. As a consequence, ARF proteins are released which turn on the transcriptional regulation of downstream genes [23]. However, the interaction between Aux/IAA and ARF proteins during the auxin-signaling pathway could regulate the expression of downstream genes has remained uninvestigated so far.

The functional characterization of *Aux/IAA*-encoding genes has been substantiated through mutants and overexpression studies in *Arabidopsis thaliana*. For example, the loss of function of *atiaa3*/*shy2* and *atiaa28* mutations results in defective lateral root formation [24, 25]. *AtIAA12* and *AtIAA18* can regulate embryo apical conformation and root meristem formation by inhibiting *AtARF5* activity [26, 27]. In addition, *SlIAA27* controls the biosynthesis of strigolactone through the regulation of *NODULATION SIGNALING PATHWAY1* (*NSP1*), therefore complementing the defective phenotype of mycorrhizal roots [28]. Recent studies have indicated that *Aux/IAA* genes are extensively involved in plants’ responses to abiotic stress. For instance, it has been discovered that the expression level of the sorghum *SbIAA1* gene is significantly induced under salt and drought stress [29]. In *Arabidopsis*, loss of *AtIAA5*/*6*/*19* resulted in reduced Glucosinolates (GLS) levels, thus resulting in reduced drought tolerance. In addition, all three single mutants, as well as the *iaa5/6/19* triple mutant, were hypersensitive to drought when compared to the Wild Type (WT) [30]. Auxin has been reported to negatively regulate Aluminum (Al) resistance by altering the expression and distribution of Al-sensitive protein 1 in *Arabidopsis* root cells, where co-treatment of IAA with Al significantly reduced Al content in root tips [31]. In rice, *OsIAA6* may trigger an auxin-mediated drought response by regulating the expression of auxin synthesis genes, thereby improving drought tolerance [32]. Under drought and salt stress, the expression level of the *OsIAA20* gene was enhanced, and overexpression of the gene significantly improved drought and salt tolerance in rice [33]. The *TaIAA15-1A* gene in wheat enhanced drought tolerance in brachypodium by regulating ABA-related genes and increasing antioxidant stress capacity [34]. Therefore, the identification of *Aux/IAA* genes and their functions is highly indispensible for growth and development, as well as improvement of crop resistance under different environmental conditions.

White clover (*Trifolium repens* L.) is an important forage legume and excellent lawn plant with high nutritional value and palatability. The lack of validated molecular markers and genetic maps for the white clover genome makes it difficult to perform genetic analysis and functional annotation [35–37]. However, whether Aux/IAA family members are involved in different biological processes of white clover has remained unclear so far. In this study, we identified and characterized 47 *Aux/IAA* genes in the white clover genome and analyzed their gene structure, phylogenetic relationships, chromosomal localization, motif patterns, cis-acting elements, and expression patterns under Al, drought, and salt stresses. Finally, we verified that *TrIAA18* interacted with *TrARF5* using a yeast two-hybrid. We also examined the positive effects of heterologous yeast expression and overexpression of *TrIAA18* in *Arabidopsis* under Al, drought and salt stress. These findings will provides better understand about the role and evolution of *Aux/IAA* genes and further supporting the study of other herbaceous *Aux/IAA* genes.

## Results

### Identification of *TrIAA* members in *T. repens*

A total of 47 *IAA* genes were identified and confirmed in the white clover genome based on the Aux domain (PF02309). According to the location of these genes on the chromosome, they are tentatively named *TrIAA1*-*TrIAA47*. The amino acid lengths of all *TrIAA* genes were 135–942 aa. Relative Protein Isoelectric points (PI) ranged from 5.16 to 10.08. The Molecular Weight (MW) ranged from 15.20 kDa to 105.17 kDa. In addition, based on subcellular localization predictions, 38 TrIAA proteins were localized to the nucleus, 8 TrIAA proteins were localized to chloroplasts, and 1 TrIAA protein was localized to the cell membrane. The diversity of subcellular localization implies a variety of biological functions within members of the white clover *IAA* gene family [38]. All details are shown in Table S1.

### Phylogenetic analysis of IAA genes in *T. repens*

**Fig. 1:**
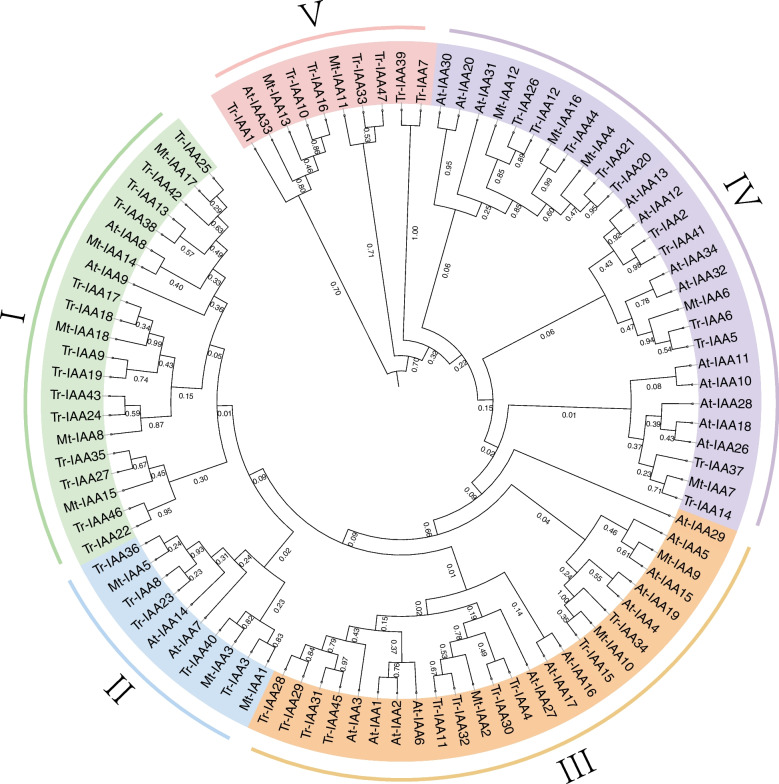
Phylogenetic analysis representing the relationship between IAA proteins in white clover, *Arabidopsis*, and *M. truncatula*. IAA subgroups (classifications I, II, III, IV, V) are marked with different colors. The phylogenetic tree was drawn using the neighbor-joining (NJ) method with a bootstrap value of 1000

To explore the evolutionary relationships of *TrIAA* genes with *IAAs* of other species, a phylogenetic tree was constructed using 29 *AtIAAs* and 18 *MtIAAs* from *Arabidopsis* and *Medicago truncatula* (Fig. 1, Table S2). According to the homology and classification of *Arabidopsis* and *M. truncatula IAA* genes, these IAAs were categorized into five clades in the phylogenetic tree: clade I (2 AtIAAs, 5 MtIAAs, and 14 TrIAAs), clade II (2 AtIAAs, *3* MtIAAs, and 5 TrIAAs), clade III (12 AtIAAs, 3 MtIAAs, and 10 TrIAAs), clade IV (12 AtIAAs, 5 MtIAAs, and 11 TrIAAs), and clade V (1 AtIAA, 2 MtIAAs, and 7 TrIAAs).

### Gene structure, motif, and cis-element analysis of *TrIAA* genes

**Fig. 2:**
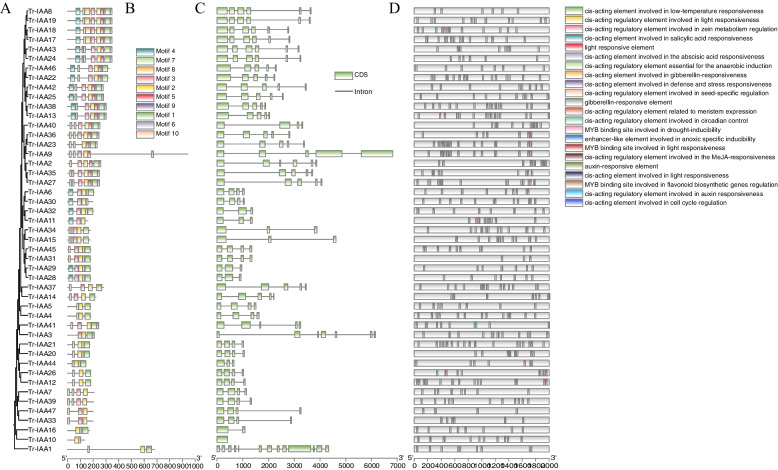
Phylogenetic relationships, motif patterns and gene structures of the white clover *IAA* gene family. **A** Phylogenetic tree of the white clover *IAA* gene family; **B** The 10 conserved IAA protein motifs are represented by different colored squares; **C** The structures of introns and exons are indicated by black lines and green boxes, respectively; **D** Characterization of cis-acting elements in the promoter region of the *TrIAA* genes

We also established a phylogenetic tree of TrIAAs (Fig. 2A). Ten different conserved motifs were identified using the MEME tool. Among the 47 TrIAA, only motif 2 was common, indicating that this structural domain was highly conserved in the IAAs family (Fig. 2B). This was followed by motif 1, motif 3, and motif 4 being relatively conserved in the IAAs family. Furthermore, gene structure analysis showed that the number of introns in the 47 *TrIAA* genes ranged from 0 to 12, with no introns in *TrIAA10*. The number of exons was 1 to13, and genes clustered together generally contained similar structures (Fig. 2C). Interestingly, *TrIAA23* has 5 exons and exhibited the longest gene length, possibly caused by a mutation in the gene [39].

To further understand the potential regulation of *TrIAA* gene expression, the 2000-bp promoter sequence of *TrIAAs* was analyzed using PlantCARE. The results showed that cis-acting elements for light response, hormone, stress, and developmental response were widely predicted in 47 *TrIAA* promoters (Fig. 2D, Table S3). Among them, the largest proportion comprised light-responsive cis-acting regulatory elements (TCCC-motif). Among the hormone category, abscisic acid (ABRE), Methyl jasmonate (TGACG-motif), auxin (AuxRR-core), salicylic acid (TCA), and gibberellin (GARE_motif) responsive cis-acting elements were dominant. Elements involved in stress mainly include low temperature response (LTR), drought-inducibility (MBS), and anaerobic induction (ARE). In addition, zein metabolism regulation (O^2−^ site) was the most common element in the *TrIAAs* promoter, followed by meristem expression factors (CAT_box), seed-specific regulation (RY_element), circadian control, and regulation of flavonoid biosynthesis genes (MBSI). These results suggest that TrIAAs may respond to a wide range of hormones, growth and development, and stresses.

### Chromosomal distribution and synteny analysis of *IAA* genes in white clover

**Fig. 3:**
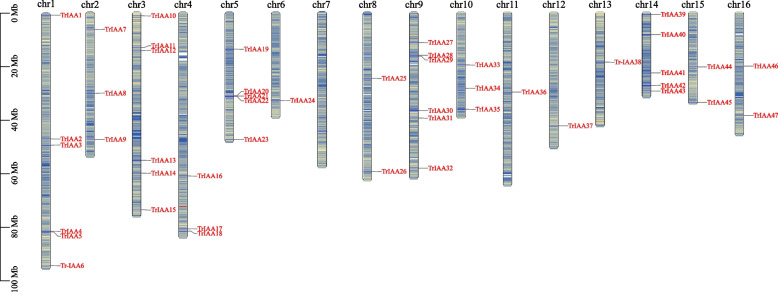
Distribution of the 47 *TrIAA* genes on 16 chromosomes. Vertical bars indicate chromosome density, and the scale on the left indicates chromosome length

To better understand the distribution mechanism of *TrIAA* gene chromosomes, a chromosome map of TrIAA was constructed using TBtools. The results showed that all chromosomes had *TrIAA* gene except chromosome 7, which lacked *TrIAA* gene (Fig. 3). Among them, chromosomes 1, 3 and 9 had six *TrIAA* genes, while chromosome 6, 11, 12 and 13 exhibited only one *TrIAA* gene, respectively.

**Fig. 4:**
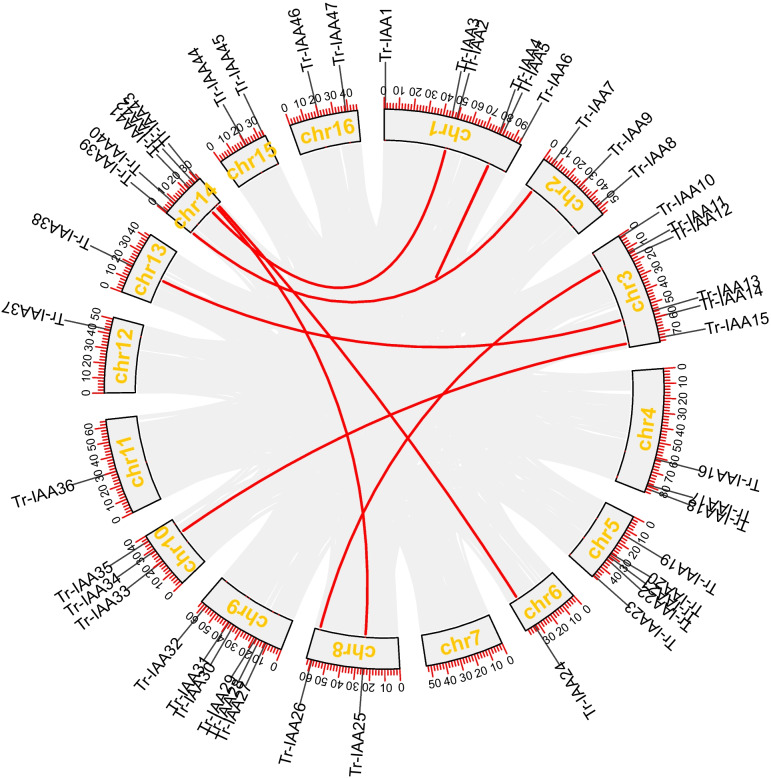
Distribution and covariance analysis of IAA in the white clover genome. The red line represents the covariance between *TrIAA*

**Fig. 5:**
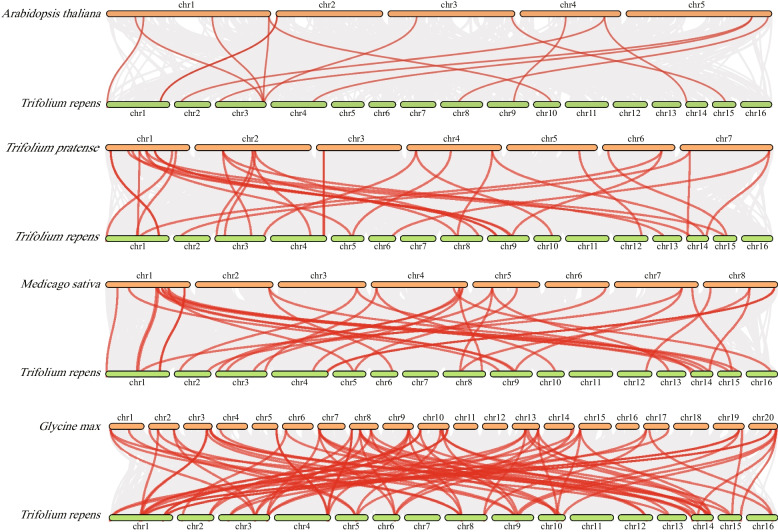
Collinearity analysis of *IAA* genes between white clover, *Arabidopsis*, red clover, *M. truncatula* and soybean. Gray lines represent blocks of covariance within the white clover and other plant genomes, and red lines represent covariant *IAA* gene pairs

The *TrIAA* gene was further analyzed for collinearity (Fig. 4). The results showed that 7 pairs of *TrIAA* genes were in segmental duplication (SD), including *TrIAA3*/*TrIAA41*, *TrIAA7*/*TrIAA39*, *TrIAA11*/*TrIAA26*, *TrIAA13*/*TrIAA38*, *TrIAA15*/*TrIAA34*, *TrIAA24*/*TrIAA43*, and *TrIAA25*/*TrIAA42* (Table S4). In addition, one pair of tandem duplications (TD) was found on chromosome 1 (*TrIAA4*/*TrIAA5*) (Table S4). It is suggested that SD is the main amplification mechanism of the *TrIAA* gene family [40, 41]. We also investigated *TrIAA* homologous gene pairs in white clover and *Arabidopsis*, red clover, *M. truncatula*, and soybean (Fig. 5). The results showed that there were 11 homology pairs (23.4%) between white clover and *Arabidopsis*, 30 homology pairs (63.8%) with red clover, 32 homology pairs (68.1%) with *M. truncatula*, and 36 homology pairs (76.6%) with soybean, respectively (Table S5). The high homology of white clover IAA with soybean indicates that IAA protein sequence and function are highly conserved and more closely related to soybean.

### Expression patterns of *TrIAA* genes under abiotic stresses and subcellular localization

**Fig. 6:**
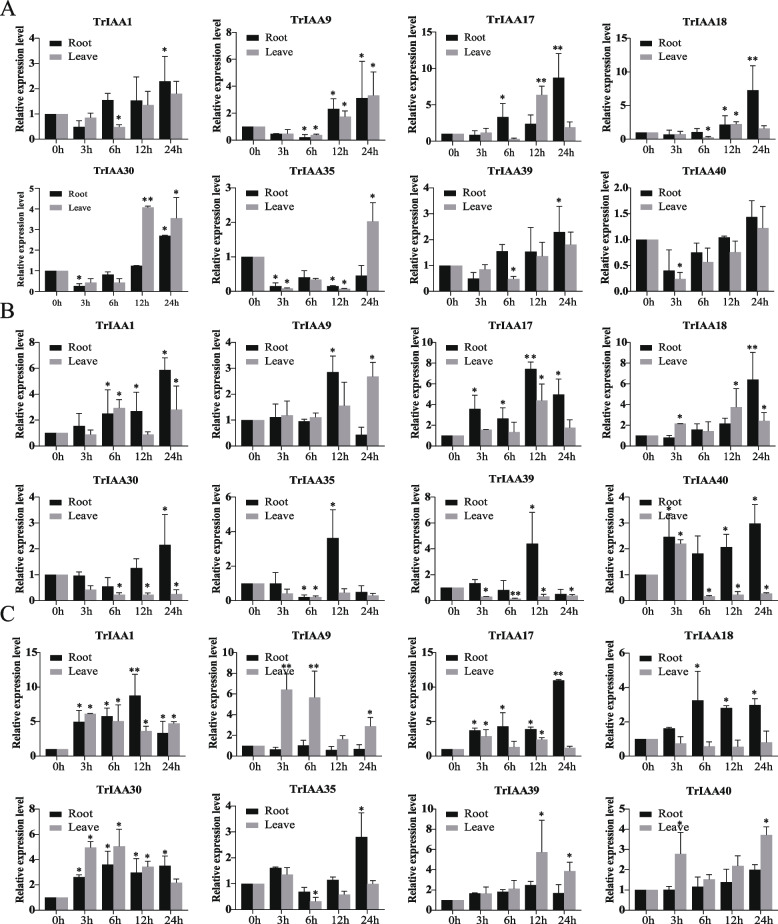
Expression levels of eight *TrIAA* genes in roots and leaves in response to aluminum (**A**), drought (**B**), and salt (**C**) stress. Error lines indicate the mean ± standard deviation (SD) between three replicates. * denotes significant differences compared with the untreated control (Student's t test, * *P* < 0.05; ** *P* < 0.01)

To further investigate the transcriptional changes of TrIAA in response to different abiotic stress treatments, we randomly selected 8 TrIAAs representing the three branches of TrIAAs in the phylogenetic tree. We analyzed their expression patterns in roots and leaves using qRT-PCR at different time intervals under various stress conditions (Fig. 6). Under Al (pH = 4.5) stress, *TrIAA17* and *TrIAA18* were expressed at significantly higher levels in roots than the control, especially at 24 h. *TrIAA17* and *TrIAA30* were highly expressed in leaves compared to the control. Under drought stress, *TrIAA1*, *TrIAA17* and *TrIAA18* were highly expressed at 24 h compared to the control, whereas *TrIAA30*, *TrIAA35*, *TrIAA39* and *TrIAA40* showed a gradual decrease in expression in leaves. However, the expression of most *TrIAA* was up-regulated under salt treatment. Among them, *TrIAA1* was significantly elevated in both roots and leaves, followed by *TrIAA17* and *TrIAA18*. In addition, *TrIAA9* was preferentially expressed in leaves. The results suggest that *TrIAA* gene family members can respond to different stress conditions.

**Fig. 7:**
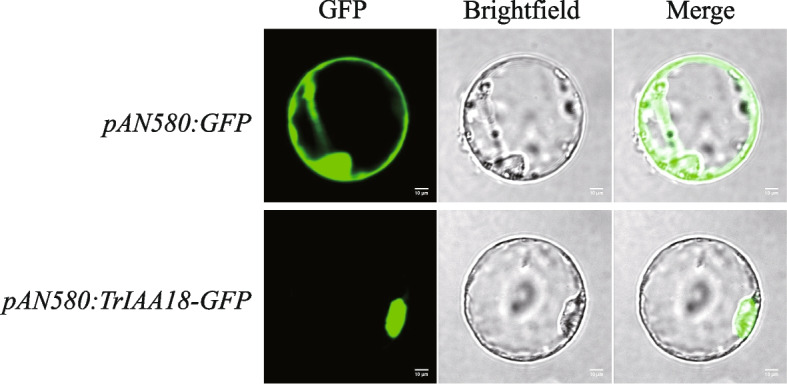
Subcellular localization of *TrIAA18* protein. *TrIAA18*-GFP were transiently expressed in rice protoplasts. Scale bars = 10 μm

According to the qRT-PCR results, *TrIAA18* was selected for subcellular localization analysis because it was strongly induced by AlCl_3_, PEG and NaCl treatments. The full-length CDS of *TrIAA18* was cloned from ‘Ladino’ and then used to construct the corresponding transient vector for injection into rice protoplasts. As shown in Fig. 7, the *TrIAA18*-GFP fusion protein was expressed in the transformed protoplasts and localized in the nucleus, demonstrating the role of *TrIAA18* as a transcription factor regulating relevant biological processes.

### *TrIAA18* expression enhances abiotic stress tolerance in yeast and interacts with *TrARF5*

The coding sequence of *TrIAA18* was cloned and expressed in *Saccharomyces cerevisiae* strains BY4741 and INVSC1, respectively. Positive BY4741 yeast cells were plated on Synthetic Galactose Minimal Medium without Uracil (SG-Ura) containing 2 mM AlCl_3_, and positive INVSC1 yeast cells were plated on SG-Ura containing 2 M D-Mannitol and 1.5 M NaCl. Yeast overexpressing *TrIAA18* grew better than the wild type under Al, drought, and salt stress conditions (Fig. 9A). When the bacterial solution was diluted to 10^–threefold^, wild-type yeast could not survive at 1.5 M NaCl, but transgenic yeast overexpressing *TrIAA18* could grow well under these conditions (Fig. 9A). These results suggest that *TrIAA18* may respond positively to abiotic stress.

**Fig. 8:**
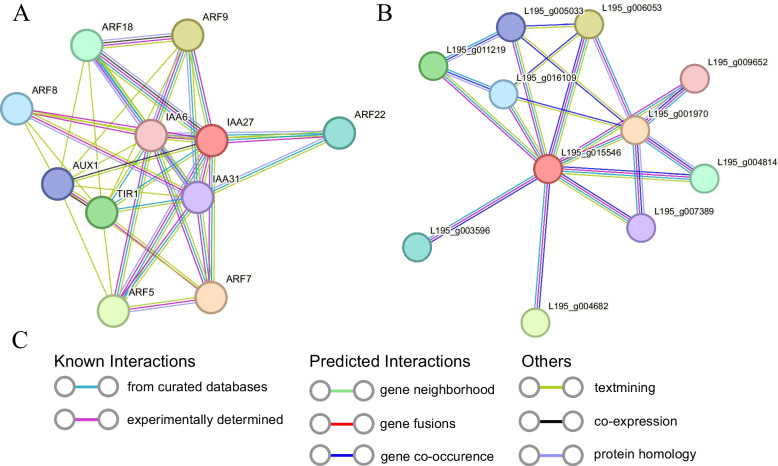
Protein interaction network of *TrIAA18* based on immediate homologs in *Arabidopsis* (**A**) and red clover (**B**). **C** Different protein–protein associations are listed

**Fig. 9:**
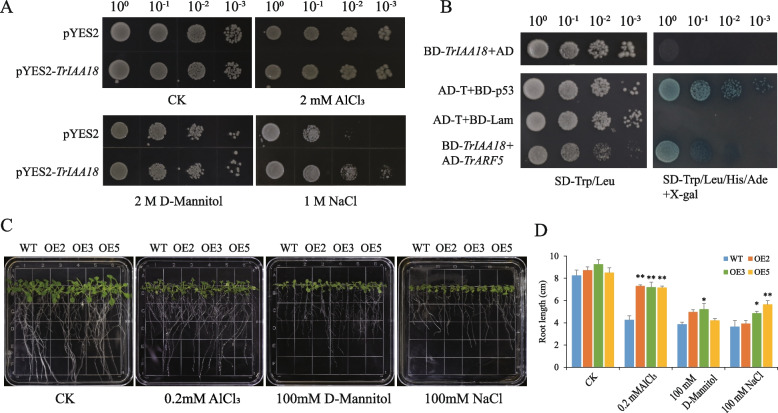
Overexpression of *TrIAA18* increases aluminum, drought, and salt tolerance in yeast and *Arabidopsis*. **A** Growth of BY4741 and INSVC1 yeast transformed with *TrIAA18*-containing pYES2 and the empty vector pYES2. **B** Yeast two-hybrid assay of *TrIAA18* and *TrARF5*. Transcriptional activity assay of *TrIAA18* was measured on selection medium, and then *TrIAA18* fused with GAL4-BD and co-transformed with *TrARF5* fused with GAL4-AD into AH109 and grown on selection medium. **C** Root length phenotypes of Wild-Type (WT) and *TrIAA18*-OE plants under normal conditions (CK), 0.2 mM AlCl_3_, 100 mM D-Mannitol, and 100 mM NaCl treatments. **D** Total root length of seedlings of WT and *TrIAA18*-OE plants. The asterisks indicate the significant differences compared with the WT plants (Student's test, * *P* < 0.05; ** *P* < 0.01)

To explore potential roles of *TrIAA18*, protein–protein interaction was predicted against the protein databases of a model plant (*Arabidopsis*) and a legume plant species (red clover) using STRING (https://string-db.org/). As shown in Fig. 8A, *AtIAA27* (a *TrIAA18* homolog) may interact with three AUX/IAA proteins (IAA6, IAA31, and AUX1), six ARF proteins (ARF5, ARF7, ARF8, ARF9, ARF18, and ARF22), and the TIR1/AFB protein (TIR1). Similarly, interactions were predicted between homologs of *TrIAA18* in red clover (*L195_g015546*) and seven ARF proteins. Since high protein identity exists between *TrIAA18* and *AtIAA27* as well as *L195_g015546* (56.9% and 96.4%, respectively; Fig. 8B), *TrIAA18* may be involved in abiotic stress response through interaction with ARF proteins. Here, we performed a yeast two-hybrid assay to verified that the *TrIAA18* was not transcriptionally active and cotransformed AH109 yeast with *TrARF5*. As shown in Fig. 9B, the cotransformed positive yeast turned blue on the defective medium (SD-Trp-Leu-His-Ade) containing x-α-gal. The results showed that *TrIAA18* interacted with *TrARF5.*

### *TrIAA18* overexpression in *Arabidopsis* improves abiotic stress tolerance

To further investigate the function of *TrIAA18* during stress response, three independent homozygous T3-generation transgenic *Arabidopsis* plants were studied under different stress treatments (Fig. 9C, D). Phenotypic analysis indicated that under 0.2 mM Al treatment, the root lengths of all three *TrIAA18*-OE plants were significantly longer than those of WT plants (Fig. 9D). Under 100 mM D-Mannitol treatment, only *TrIAA18-*OE3 plants had significantly longer root lengths compared to WT plants (Fig. 9D). Under 100 mM NaCl treatment, *TrIAA18-*OE3 and *TrIAA18-*OE5 strains had significantly or highly significant root lengths than WT plants (Fig. 9D). These results indicate that transgenic plants are more resistant to Al, D-Mannitol and salt stresses compared to WT plants.

## Disscussion

The *Aux/IAA* gene family is a family of plant-specific transcription factors that play important roles in growth factor signaling and various physiological processes [2, 4]. White clover is an excellent forage legume with high protein and good palatability, but its growth and development are often susceptible to various environmental adversities [36]. While extensive research has been conducted on the *Aux/IAA* genes of model plants like *Arabidopsis* and rice, the exploration of these genes in white clover remains limited. Therefore, the study of the *Aux/IAA* gene in white clover will not only help to reveal its growth hormone signaling mechanism but also help to explore its stress tolerance mechanism.

In this study, using annotated files and newly released white clover genome data, 47 *Aux/IAA* genes were identified by homology comparison and structural domain analysis and designated as *TrIAA1-TrIAA47* [42]. Compared with 29 in *Arabidopsis* [43] and 18 in *M. truncatula* [44], the number of *Aux/IAA* genes was significantly increased in white clover and showed extensive variations in Open Reading Frame (ORF) length, predicted MW, and PI. These changes suggest that different TrIAA proteins may function in different microenvironments [5, 45, 46]. According to phylogenetic analyses, most TrIAAs were closely related to AtIAAs and MtIAAs. *AtIAA18* was found to be associated with lateral root formation in *Arabidopsis*. A previous study showed that the *AtIAA5/6/19* deletion resulted in reduced GLS levels and decreased drought tolerance, suggesting that Aux/IAA proteins could regulate drought tolerance in *Arabidopsis* by regulating GLS levels [30]. Taken together, these data suggest that the *TrIAA* genes, which are closely related to *AtIAA* and *MtIAA*, may also be involved in growth and development and response to abiotic stresses.

AUX/IAA proteins typically have four conserved structural domains that define members of the gene family, referred to as I, II, III, and IV [10–12]. A total of 16 TrIAA proteins contain four structural domains, while other proteins were missing at least one structural domain. Analysis of the exon/intron structure of the gene showed that the number of exons/introns in *TrIAA* was highly variable, ranging from 1 to 13, similar to *Arabidopsis* [44, 47]. Taken together, the structural features of TrIAA proteins within each subfamily are relatively conserved, and the structural variability among different *TrIAA* genes may contribute to the functional diversity of the *AUX/IAA* gene family. In addition, cis-regulatory elements in the promoter region play important roles in plant growth and development, secondary metabolism and response to adversities [48, 49]. In this study, most *TrIAA* gene promoters contained growth hormone-responsive (AuxRE/TGA-element) cis-elements or their variants [50]. Some *TrIAA* gene promoters have been reported to contain cis-elements involved in abiotic stress response, for example, LTR is involved in low temperature response [51], MBS plays a role in drought- inducibility [52, 53], ARE is involved in anaerobic induction, and TC_rich_repeats are involved in defense and stress response [54]. In conclusion, the different types of cis-elements on the promoters of the *TrIAA* gene family reflect the complexity and diversity of gene expression regulation and evolution.

Gene duplication events promote rapid expansion and evolution of species gene families by doubling chromosomes. A total of 8 gene pairs with gene duplication events (7 segmental duplication gene pairs and 1 tandem duplication gene pair) were identified in white clover, suggesting that the expansion of the *TrIAA* gene family may have occurred primarily through segmental duplication [40, 41]. The covariance of *IAA* genes in white clover, *Arabidopsis*, red clover, *M. truncatula*, and soybean was analyzed using TBtools (v2.019, Chengjie Chen, Guangzhou, China) software. A total of 15 TrIAA-AtIAA pairs, 34 TrIAA-MtIAA pairs, 37 TrIAA-TpIAA pairs, and 79 TrIAA-GmIAA pairs were observed in the Aux/IAA family. The results showed that white clover was phylogenetically more related to soybean than to *Arabidopsis*.

Previous studies have shown that *AUX/IAA* genes have regulatory functions in abiotic stresses. For example, *OsIAA20* confers drought and salt tolerance to transgenic rice by increasing proline and chlorophyll content via ABA signaling pathway [33]. *OsIAA6* is involved in the downward growth of the rice root system, thereby enhancing drought tolerance [32]. In this study, *TrIAA* genes showed different expression patterns in different tissues and under different stress treatments. Among them, the expression levels of *TrIAA17* and *TrIAA18* were highly expressed at different time points under Al, drought, and salt stresses, indicating the potential multiple functions of this gene. It was further verified by yeast heterologous expression and transgenic *Arabidopsis* that *TrIAA18* could enhance its stress tolerance. Our research provides insights into the expression spectrum of *TrIAAI8* under various stress conditions, but further studies are needed to elucidate the stress response mechanisms in white clover and to confirm its potential as a target for enhancing plant stress tolerance.

In addition, it has been demonstrated that AUX/IAA proteins can interact with ARF proteins to regulate the expression of downstream target genes. In wheat, *TaIAA8* was localized in the nucleus and responded to growth hormone signaling, thereby regulating lateral root growth by interacting with *TaARF21* [55]. In apple (*Malus domestica*), *MdARF13* affects anthocyanin synthesis by interacting with *MdIAA121*, which is characterized by growth hormone-induced degradation of *MdIAA121* and release of *MdARF13*, which negatively regulates anthocyanin metabolic pathways [56]. However, the regulation of plant responses to abiotic stresses by Aux/IAA and ARF through specific interactions has rarely been reported. In this study, we confirmed that *TrIAA18* could interact with *TrARF5* by yeast two-hybrid assay. It is hypothesized that *TrIAA18* may enhance stress tolerance by affecting *TrARF5*, but its regulatory function needs to be further explored in future studies. However, current research has preliminarily elucidated the response of the *TrIAA18* to abiotic stress, and it is important to recognize the potential role of other *Aux/IAA* genes in mediating stress responses. Future research could benefit from a broader examination of the *Aux/IAA* gene family, exploring the synergistic effects and regulatory networks that contribute to the plant’s resilience. Such an inclusive approach would not only enhance our understanding of the gene family’s collective response to environmental challenges but also pave the way for developing more robust stress-tolerant crops.

## Conclusion

In conclusion, 47 *IAA* gene family members were identified from the white clover genome and categorized into five classes based on their phylogenetic relationships with *Arabidopsis* and *M. truncatula* IAA. Structural analyses, including gene structure, motif composition, and homologous protein modeling, indicated that TrIAA proteins in specific subfamilies were relatively conserved. The coexistence of different cis-regulatory elements involved in developmental regulation, hormones, and stress responses in their promoter regions may be closely related to the multiple regulatory roles of TrIAAs. Collinearity analyses indicated that the expansion of the *TrIAA* gene family might have occurred primarily through segmental duplication, whereas many other duplicate gene pairs exhibited different expression profiles, suggesting their functional diversity. Finally, *TrIAA18* was shown to be involved in the positive regulation of Al, drought, and salt stress, as well as interaction with *TrARF5*, indicating that *TrIAA18* may regulate abiotic stress responses by affecting *TrARF5*. These results will provide foundation for future studies about the regulatory functions of IAA proteins in forage legumes exposed to abiotic stresses.

## Materials and methods

### Plant materials and treatments

The white clover cultivar ‘Ladino’ used in this study was purchased from Evergeen International Co., Ltd. Seeds of Ladino were first germinated in plastic containers with quartz sand and sterile ddH_2_O (22/20 ℃, 16/8 h, light/dark). After one week, seedlings were allowed to grow in Hoagland’s nutrient solution for the next 23 days. Later, one-month-old white clover seedlings were subjected to Al stress (2 mM AlCl_3_), drought stress (15% PEG), and salt stress (100 mM NaCl), respectively. Leaves and root samples were collected at 0, 3, 6, 12, and 24 h after stress treatments. Samples for all time points were taken with three independent biological replicates, frozen in liquid nitrogen, and stored at -80 ℃.

### Identification and bioinformatic analysis of *TrIAA* genes

White clover genome resource information and annotation files were provided by NCBI (https://www.ncbi.nlm.nih.gov/datasets/genome/GCA_030408175.1/) and Stig Uggerhøj Andersen, Aarhus University [42]. To identify all members of the IAA family, a Hidden Markov Model of the IAA domain (PF02309) was downloaded from the Pfam Protein Family Database (http://Pfam.xfam.org/), and the *IAA* genes were then identified from the white clover genome using HMMER 3.0 [57, 58]. Subsequently, the potential *TrIAA* was further identified in the NCBI database (http://www.ncbi.nlm.nih/gov/structure/cdd/wrpsb.cgi) through conserved structural domains. Physicochemical properties such as amino acid length, theoretical isoelectric point, and molecular weight of the TrIAA protein were analyzed using the online Prosite ExPASy (https://www.expasy.org/) and SMART (http://smart.embl.de/smart/batch.pl) servers [59, 60]. The subcellular localization of TrIAA proteins was predicted using BUSCA (http://busca.biocomp.unibo.it/) [61].

### Phylogenetic analysis

IAA protein sequences from *Arabidopsis* and *M. truncatula* were downloaded from Ensembl Plant (http://plants.ensembl.org/index.html) [62]. All IAA protein sequence comparisons were performed using clustalW (https://www.ebi.ac.uk/Tools/msa/clustalo/), and the output data were saved in Mega format [63]. The phylogenetic tree was then constructed with MEGA6 software using the Neighbor-Joining (NJ) method with 1000 bootstrap replicates [64]. The phylogenetic tree was visualized and optimized using iTOL (https://itol.embl.de/) [65].

### Gene structure, motif and cis-element analysis

The exons and introns of each *TrIAA* gene were obtained from the white clover genome annotation file. Conserved protein motifs were predicted using the MEME Suite web server (https://meme-suite.org/) with a maximum number of 10 motif sets and an optimal width of 5 to 200 amino acids [66]. The exons and introns of the *TrIAA* gene, as well as the conserved motifs and structural domains of the TrIAA protein, were visualized and analyzed using TBtools software. Finally, the PlantCARE database (http://bioinformatics.psb.ugent.be/webtools/ plantcare/html/) was used to analyze cis-acting elements in genomic DNA sequences 2000 bp upstream of the transcription start site of each *TrIAA* gene [67].

### Chromosomal locations, gene duplication, and syntenic analysis

The chromosomal location of each *TrIAA* gene was retrieved from the GFF3 file of the white clover genome and visualized using TBtools. The Multiple Covariance Scanning Toolkit (MCScanX, default parameters) was utilized to analyze the duplication events of the *TrIAA* gene [68]. TBtools software was used to construct synchronization maps of white clover with four other plants (*Arabidopsis*, red clover, *M. truncatula*, and soybean).

### RNA extraction and qRT-PCR analysis

Total RNA was extracted from leaf and root samples using the HiPure Plant RNA Mini Kit (Magen, Guangzhou, China). cDNA was synthesized using the Monad MR00101 kit (Monad, Suzhou, China). cDNA was then analyzed on a CXF Connect™ Real-Time System (Bio-RAD) using the ABM®Evagreen 2 × QPCR Master Mix (ABM, Canada), following the manufacturer’s protocol. qRT-PCR analyses were performed according to the manufacturer’s protocol. The *ACTIN* gene was used as an internal reference. Gene expression was quantified using the 2^−∆∆CT^ method [69]. Three biological replicates were used for each data point. The primers used in this study are listed in Table S6.

### Subcellular localization analysis of *TrIAA18*-GFP protein

The CDS of *TrIAA18* was fused to the PAN580 subcellular localization vector, with Green Fluorescent Protein (GFP) at the N-terminus. Rice protoplasts were prepared and transformed as described previously [70], and then stained using a confocal laser scanning microscope (ZESS LSM880) to observe the green fluorescence signals.

### Heterologous expression of *TrIAA18* in yeast

The CDS sequence of the *TrIAA18* was amplified using homology arm primers with restriction endonuclease cleavage sites *NdeI* and *BamHI*. The *TrIAA18* was then ligated into the pYES2 vector using the MonClone™ Single Assembly Cloning Mix (Monad, Suzhou, China) and transformed into *Escherichia coli* DH5α using heat excitation. *Saccharomyces cerevisiae* strains, BY4741 and INVSC1 were further transformed with the pYES2-*TrIAA18* recombinant vector using the Yeast Transformation Kit (Coolaber, Beijing, China). Yeast cells were transformed in the presence of uracil-free glucose (SD-Ura) agar medium for growth. SG-Ura agar medium without and containing 2 mM AlCl_3_ (pH = 4.5), 2 M D-Mannitol, and 1.5 M NaCl was prepared as described in the literature [71]. Positive transformants were identified and incubated with SG-Ura liquid medium containing galactose until OD_600_ = 1, then serially diluted (OD_600_ = 1, 10^–1^, 10^–2^, 10^–3^, 10^–4^). 8 μL of each dilution was dropped into a different medium and incubated at 30 °C for 3–5 days.

### Yeast two-hybrid assay

The *TrIAA18* protein sequence was submitted to the STRING website (http://string-db.org). Direct homologs of *Arabidopsis* and red clover were selected as references for constructing the reciprocal network [72]. Yeast two-hybrid assays were performed using the Yeastmaker Yeast Transformation System 2 (Clontech, San Francisco, CA, USA). The coding sequences of *TrARF5* and *TrIAA18* were cloned into the pGADT7 and pGBKT7 vectors, respectively. The pGADT7-*TrARF5* and pGBKT7-*TrIAA18* plasmids were transformed into *Saccharomyces cerevisiae* strain AH109 using the lithium acetate method [73]. Yeast cells were cultured on defective (-Leu/-Trp) medium according to the manufacturer's instructions. Positive yeast cells were plated onto defective (-Leu/-Trp/-His/-Ade) medium containing X-gal to verify their interaction.

#### Genetic transformation of *Arabidopsis* and treatment under abiotic stress

The cDNA of *TrIAA18* with *EcoRI* and *BamHI* homology arm sequences was cloned into the overexpression vector pCAMBIA1301-GFP (pCAMBIA1301-GFP-*TrIAA18*). The pCAMBIA1301-GFP-*TrIAA18* plasmid was transformed into *Agrobacterium tumefaciens* strain GV3101, and transgenic *Arabidopsis* plants were obtained using the flower maceration method [74]. T1 seeds obtained from the transformants were germinated on MS medium containing thaumatin, screened for resistant plants, and functionally verified with T3-banded pure sum lines. T3-generation seeds were germinated flat into 1/2 MS medium containing 0.2 mM AlCl_3_ (pH = 4.5), 100 mM D-Mannitol, and 100 mM NaCl for 10 days, and root length was determined.

#### Statistical analysis

All experimental data were expressed as the mean SE of at least three biological replicates. ANOVA with *P* < 0.05 or *P* < 0.01 was performed using SPSS 25 software to identify significant or very significant differences, respectively.

### Supplementary Information

**Additional file 1:** **Table S1. **Information and physicochemical properties of the *TrIAA* genes. **Table S2. **Gene IDs and corresponding nomenclature in *TrIAAs*/*AtIAAs*/*MtIAAs*.**Table S3. **Prediction of cis-regulatory elements in promoter regions of *TrIAAs***. Table S4. **Segmentally and tandemly duplicated *TrIAA* gene pairs. **Table S5. **Orthologous relationships of *IAA* genes between *Trifolium repens* and ten representative plants.** Table S6. **The specific primers of *TrIAA* genes for qRT-PCR.

## Data Availability

All of the datasets supporting the results of this article are included within the article and its additional files.
